# EGFR-TKI原发性耐药的分子机制及其进展——附2例病例分析

**DOI:** 10.3779/j.issn.1009-3419.2019.01.10

**Published:** 2019-01-20

**Authors:** 美蓉 刘, 凡路 孟, 晴 马, 立彦 顾, 殿胜 钟

**Affiliations:** 300052 天津，天津医科大学总医院肿瘤内科 Department of Medical Oncology, Tianjin Medical University General Hospital, Tianjin 300052, China

**Keywords:** 肺肿瘤, EGFR-TKI, 原发性耐药, Lung neoplasms, EGFR-TKI, Primary resistance

## Abstract

酪氨酸激酶抑制剂(tyrosine-kinase inhibitor, TKI)类药物已经被证实对表皮生长因子受体(epidermal growth factor receptor, *EGFR*)敏感突变的晚期非小细胞肺癌(non-small cell lung cancer, NSCLC)患者有很好的疗效，优于化疗。但仍有部分敏感突变的患者出现原发性耐药。耐药的原因尚不明确，可能与*EGFR*基因的敏感突变与耐药突变共存、EGFR通路下游基因突变、MET扩增、BIM缺失多态性等因素相关。本文分享了2例原发性耐药病历并进行了原因分析。

## Abstract (en)

Tyrosine kinase inhibitor (TKI) have been proved to be effective in the treatment of advanced non-small cell lung cancer (NSCLC) patients with epidermal growth factor receptor (EGFR) sensitive mutation, which is superior to chemotherapy. However, there are still some patients with sensitive mutations have primary drug resistance. It may be related to the coexistence of susceptible and resistant mutations of EGFR gene, downstream mutations of EGFR pathway, MET amplification and BIM deletion polymorphism. We present 2 cases of primary drug resistance and analyze the reasons.

随着酪氨酸激酶抑制剂（tyrosine-kinase inhibitor, TKI）类药物的研发及在临床的广泛应用，表皮生长因子受体（epidermal growth factor receptor, *EGFR*）敏感突变的晚期非小细胞肺癌（non-small cell lung cancer, NSCLC）患者的客观反应率（objective response rate, ORR）、无进展生存时间（progression-free survival, PFS）和总生存时间（overall survival, OS）都较单纯化疗有了显著的提高。但仍有少部分*EGFR*敏感突变患者，服用TKI后无效。有学者将服用TKI后PFS < 90 d的患者称为原发性耐药^[1]^。目前，我们对原发性耐药的分子机制知之较少，本文结合我科两个原发性耐药病例进行分析。

## 1 病例分析

### 1.1 病例一

男性，49岁，2016年1月无诱因出现左下肢近端疼痛，在当地骨科医院行腰椎影像学检查，考虑“腰椎间盘突出”，给予对症止痛治疗，症状无明显缓解。后因脐周胀痛，查全腹部增强计算机断层扫描（computed tomography, CT）：“考虑急性胰腺炎，肝内发现多发低密度结节影，性质待定，左侧肾上腺结节，考虑转移可能性大；左侧髂骨、髋臼、耻骨联合左侧面及左侧耻骨支局部骨质破坏，考虑转移”。胸部CT显示：“右上叶肿块，纵隔内多发轻度增大淋巴结，两侧胸腔积液”。头部强化磁共振成像（magnetic resonance imaging, MRI）检查：“左侧额叶、顶叶皮层异常强化灶，考虑转移瘤；右侧额叶异常强化灶，不除外转移”；骨扫描（emission computed tomography, ECT）：“左侧髋骨、股骨近端及右侧肩胛骨异常示踪剂浓集区，考虑转移”；经皮肺穿刺活检，病理诊断：“非小细胞癌，倾向于腺癌，免疫组化染色示癌细胞呈CK7、NaspsinA、TTF-1和CK9阳性，CK20阴性”。基因检测：“*EGFR* 19外显子缺失突变，*ALK*基因无重排，*KRAS*无突变”。给予厄洛替尼150 mg *qd*，1个月后，影像学见右肺上叶肿块及左侧肾上腺肿块增大，新发小脑转移，评估为疾病进展（progressive disease, PD）（图 1），改为紫杉醇150 mg d1、d8，联合卡铂700 mg d1，每3周1次，化疗2周期，评估肺部病变仍为进展。建议患者再次活检行基因检测，患者放弃治疗自动出院，于2016年7月去世，OS 5个月。

**1:**
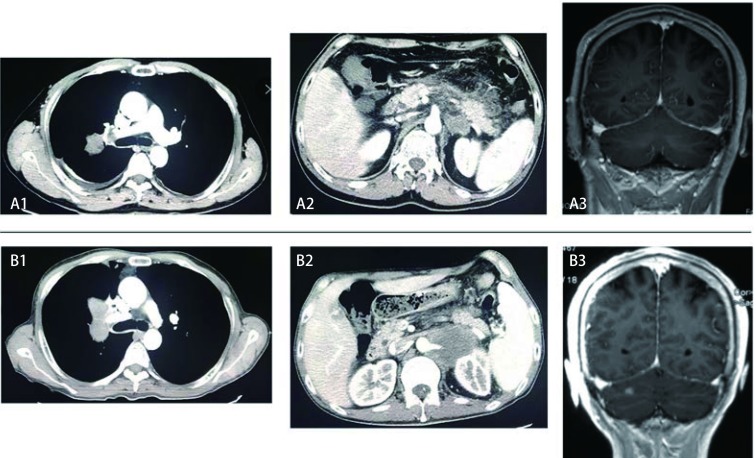
给予厄洛替尼前后肺、腰及脑部的影像学变化。A:治疗前；B：治疗1个月后。 Imaging changes of lung, waist and brain before and after treatment with erlotinib. A: Before treatment; B: One month after treatment.

### 1.2 病例二

男性，58岁，因“反复咳嗽、咳痰5个月，痰中带血2个月”，2016年7月查胸部增强CT：“左肺下叶不规则分叶状软组织肿块，肿块紧贴邻近降主动脉及右下肺静脉；左肺门、纵隔内多发肿大淋巴结”；正电子发射型计算机断层显像（positron emission computed tomography, PET）-CT：“左肺下叶后基底段软组织肿块影，代谢异常增高；双颈部及胸部多发增大淋巴结、肝右叶后下段低密度结节影及体部多发骨质密度不均，代谢异常增高，考虑为转移”。头部强化MRI未见转移灶。支气管镜病理：“（左下叶后基底段-亚段开口）低分化癌”。经皮肺穿刺活检病理：“（左下肺）NSCLC，倾向腺癌”。基因检测：“*EGFR* 21外显子L858R突变阳性；*ALK*基因无重排；*KRAS*无突变”。患者参加埃克替尼双倍剂量治疗21外显子突变晚期NSCLC的临床研究，随机到双倍剂量组，口服埃克替尼250 mg每日3次治疗，1个月后复查胸部CT平扫：患者左肺下叶分枝状肿块较之前减小，部分缓解（partial response, PR）。服药后3个月，患者咳嗽、咳痰加重，腹部及腰部疼痛明显，再次入院评估，肿瘤全面进展（图 2）。行血液基因检测，结果：“*EGFR* 21外显子L858R突变阳性，丰度8%；无T790M突变”。换用培美曲塞、顺铂联合贝伐珠单抗治疗，2周期后评估，PR；继续原方案化疗3周期后，再次评估，PD，全身多发骨转移较前增多。患者放弃治疗，于2017年8月去世，OS 12个月。

**2:**
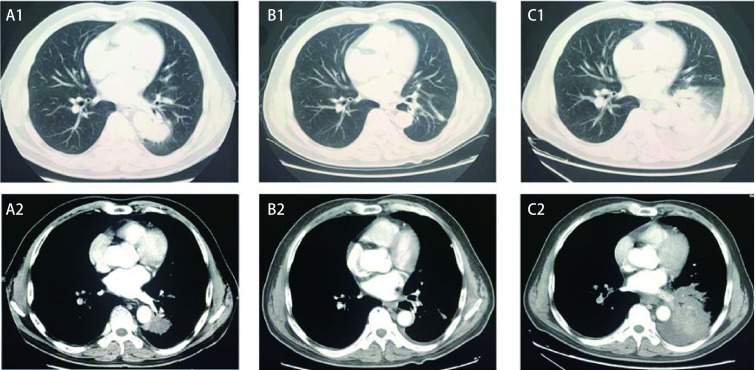
给予TKI前后腹部及肺部的影像学变化。A:治疗前；B:治疗1个月后；C:治疗3个月后。 Imaging changes of abdomen and lung before and after TKI. A: Before treatment; B: 1 month after treatment; C: 3 months after treatment.

## 2 讨论

目前，我们对EGFR-TKI原发性耐药分子机制的认知还处于探索阶段，研究表明，可能与以下分子机制有关。

### 2.1 *EGFR*基因突变诱导的耐药

*EGFR*基因的敏感突变与耐药突变共存，如20外显子插入突变、T790M突变。*EGFR* 20外显子插入突变约占*EGFR*突变的4%，多位于*EGFR*酪氨酸激酶区768-774位氨基酸，其中770位插入突变最为常见。T790M突变是位于20号外显子中第790苏氨酸（T）突变为甲硫氨酸（G）。这些耐药突变可以阻滞EGFR-TKI与EGFR靶部位结合，导致原发性耐药^[2, 3]^。在AURA 3研究中^[4]^，他们发现对于有T790M的耐药患者，奥西替尼是合适的治疗选择。此外，在BELIEF研究中^[5]^，入组的*EGFR*敏感突变NSCLC，给予厄洛替尼联合贝伐单抗治疗，亚组分析显示，同时伴有T790M突变的患者，PFS为16.0个月，提示，第一代EGFR-TKI联合抗血管生成治疗可以用于*EGFR*敏感突变伴有T790M突变的NSCLC患者。再有，Poziotinib显示出对*EGFR* 20外显子插入突变的NSCLC有效^[6]^。Poziotinib在*EGFR* 20外显子突变的细胞系Ba/F3中，IC_50_值为1.0 nmol/L，有效性是阿法替尼的40倍。在MD安德森癌症中心进行的Ⅱ期临床研究中，11例EGFR 20外显子插入突变的NSCLC患者接受Poziotinib治疗，ORR为64%，随访期6.5个月，中位OS仍未达到，使得临床医生对Poziotinib充满了期待。

### 2.2 EGFR通路下游基因突变诱导的耐药

EGFR通路下游基因，如*KRAS*、*Braf*、*PIK3CA*等同样可以发生突变。这些突变可导致RAS-RAF-MAPK和/或PI3K-AKT通路持续激活，且不受上游EGFR的调控，所以，TKI虽阻断上游通路，但下游通路仍持续活化，因此，导致EGFR-TKI原发性耐药^[7-9]^。针对这一耐药机制的治疗药物数据尚不成熟。BGB-283是RAF抑制剂^[10]^，对RAF二聚体的抑制作用增加，可同时阻断RAF和EGFR。来自中国的一项Ⅰ期临床试验中^[11]^，BGB-283对*RAF*突变的肺癌，ORR是16.7%，疾病控制率达50%。肺癌中BRAF单突变频率约为2%-4%，在一项Ⅱ期研究中^[12]^，78例经治的RAF突变晚期NSCLC患者，BRAF抑制剂达拉非尼ORR 33%，中位PFS 5.5个月，中位OS 12.7个月。6例初治患者中，4例达到PR。韩国的一项研究^[13]^纳入了136例*EGFR*敏感突变，并且接受EGFR-TKI治疗的患者，其中TKI原发性耐药的患者20例，对原发性耐药患者的组织进行了基因检测发现，*TP53*突变最为常见，占47%；*SMAD4*占19%，*DDR2* 16%，*PIK3CA* 15%，*STK11* 14%，*BRAF* 7%。发生在PI3K/Akt/mTOR通路的基因突变，TKI治疗的ORR为27%，突变组和无突变组PFS分别为2.1个月和12.8个月。提示该通路的基因发生突变，可能是原发性耐药的原因之一。

### 2.3 其他基因诱导的耐药

*MET*基因改变、间变性淋巴瘤激酶（anaplastic lymphoma kinase, ALK）重排等其他异常也可以激活EGFR-TKI原发性耐药^[14, 15]^。肝细胞生长因子受体（hepatocyte growth factor receptor, HGFR）是MET编码的蛋白质产物，与配体HGF结合后可以启动下游信号通路。在EGFR-TKI原发性耐药的腺癌细胞系中，HGF呈现出异常高表达。而棘皮动物微管相关蛋白样4（echinoderm micmtubule associated protein like 4, EML41）和ALK融合而形成*EML4-ALK*基因重排，其编码蛋白可形成非配体依赖性二聚体，活化ALK进而激活RAS-MEK-ERK，JAK3-STAT3和PI3K-AKT等信号通路。据广东省人民医院公布的对2, 632例NSCLC患者的研究^[16]^，*EGFR*和*ALK*双突变患者共16例，占0.6%。此外，胰岛素样生长因子受体（insulin-like growth factor receptor, IGFR）、层粘连蛋白（Laminin, LN1）过表达也可引起的EGFR旁路激活，导致TKI原发性耐药^[17]^。

另外，B细胞淋巴瘤/白血病-2蛋白相互作用的细胞凋亡中介物（BCL-2 interacting mediator of cell death, BIM）的缺失多态性可能也与EGFR-TKI耐药相关。一项韩国针对NSCLC患者的调查研究显示^[18]^，BIM缺失多态性为19%（61/541）。而上海肺科医院的调查显示^[19]^，12.8%（45/352）的NSCLC患者存在BIM缺失多态性，这部分患者对EGFR-TKI的ORR为25%，PFS 4.7个月；多因素分析显示，BIM缺失多态性是*EGFR*突变预后差的独立预后因子。Xia等^[20]^的研究再次验证了BIM缺失多态性与*EGFR*突变患者不良预后相关，245例NSCLC患者中BIM缺失多态性的发生率是12.24%，EGFR-TKI治疗的ORR 16%，BIM野生型ORR 91%，两组比较具有统计学意义。

### 2.4 microRNA与耐药

microRNA（miRNA）是一类由内源基因编码的长度约为22个核苷酸的非编码单链RNA分子。一项对比了EGFR-TKI原发性耐药患者血浆中miRNA水平的研究^[21]^显示，在原发性耐药组有15种miRNA下调，包括hsv2-miR-H19、hsa-miR-744-5p、hsa-miR-3196、hsa-miR-3153、hsa-miR-4791、hsa-miR-4803、hsa-miR-4796-3p、hsa-miR-372-5p、hsa-miR-138-2-3p、hsa-miR-16-1-3p、hsa-miR-1469、hsa-miR-585-3p、ebv-miR-BART14-5p、hsa-miR-769-3p、hsa-miR-548aq-5p，1种miRNA上调，hsa-miR-503-3p。上述miRNA可能通过调控MYC、CCND1等靶基因诱发EGFR-TKI原发性耐药。

文献^[1, 22]^中报道，EGFR-TKI原发性耐药的发生率为5%-20%，而*EGFR*敏感突变合并上述耐药突变的发生率均小于1%，所以上述分子机制不能完全解释原发性耐药的原因。本文中所报道的两例病例，EGFR-TKI治疗前未检测出上述耐药突变；其中第二例患者在耐药后敏感突变仍存在，也未发现相关耐药突变，这可能与我们基因检测的数量和方法有关。对这两例患者，我们仅进行了包括*EGFR*在内的3个-8个基因的检测，使用了ARMS等常规方法。NGS的方法虽然敏感度高，且可以平行进行多基因突变检测，但目前还未被指南推荐用于临床实践。近年来，血液ctDNA检测取得了很大的进展，王洁等^[23]^进行的BENEFIT研究显示，ddPCR检测的ctDNA EGFR突变指导的一线吉非替尼治疗的客观有效率为72.1%，中位PFS为9.5个月；同时利用NGS技术分析了多基因的变异，11.7%患者还携带其他基因变异，如*MET*、*ERBB2*、*KRAS*、*BRAF*、*RET*、*ROS1*、*TP53*、*RB1*、*PTEN*等；如合并其他基因突变，PFS仅3.9个月。但血液检测存在假阴性等问题，目前不能替代组织学检测，在2018年《中国*EGFR*突变血检共识》也只推荐了EGFR检测，方法为AMRS或者Super-ARMS。

总之，EGFR-TKI原发性耐药的机制与继发性耐药不同，耐药分子机制的多样性为治疗带来更多困难。在日常临床实践中，基因检测的数量及其方法均可影响对患者EGFR-TKI原发性耐药的判断和探索。
